# Diagnostic accuracy of diffusion-weighted imaging in variant Creutzfeldt–Jakob disease

**DOI:** 10.1007/s00234-023-03230-w

**Published:** 2023-10-13

**Authors:** G. Mackenzie, D. Summers, J. Mackenzie, R. Knight

**Affiliations:** 1 National CJD Research and Surveillance Unit, Centre for Clinical Brain Sciences, University of Edinburgh, Edinburgh, UK; 2 Department of Neuroradiology, Royal Infirmary of Edinburgh, Edinburgh, UK

**Keywords:** Variant Creutzfeldt-Jakob Disease, CJD, prion diseases, MRI, diffusion-weighted imaging

## Abstract

**Purpose:**

This study sought to investigate the diagnostic sensitivity of diffusion-weighted imaging (DWI) in variant Creutzfeldt-Jakob disease (vCJD), a prion disease with significant public health implications on account of its transmissibility. The importance of this research stemmed from the first neuropathologically confirmed vCJD case in a *PRNP* heterozygous individual in 2016, which displayed DWI features typical of sporadic CJD (sCJD). The case was classified as ‘probable’ sCJD in life, predominantly based on these imaging findings. While DWI has proven valuable in diagnosing sCJD, its utility in vCJD diagnosis remains unclear.

**Methods:**

DWI and Fluid-attenuated inversion recovery (FLAIR) images from probable and definite vCJD cases referred to the National CJD Research and Surveillance Unit (NCJDRSU) were independently analysed by an expert neuroradiologist. Scans were reviewed within a mixed cohort of CJD cases including definite sCJD and non-CJD controls.

**Results:**

FLAIR sequences demonstrated greater sensitivity in identifying the pulvinar sign in vCJD compared to DWI (73% vs 41%, p-value <0.001). Basal ganglia hyperintensities were more prevalent in DWI (84%) than FLAIR (64%), and cortical hyperintensities were exclusive to DWI (24%). The pulvinar sign showed a specificity of 98% for vCJD and was rare in sCJD.

**Conclusion:**

DWI showed reduced sensitivity compared to FLAIR imaging in detecting the pulvinar sign in vCJD. Conversely, DWI can more distinctively identify basal ganglia and cortical hyperintensities, thus leading to imaging patterns more characteristic of sCJD. Therefore, DWI should be cautiously interpreted in vCJD diagnosis, with axial FLAIR potentially providing a more precise evaluation of the pulvinar sign.

## Introduction

Creutzfeldt-Jakob disease (CJD) belong to a group of fatal neurodegenerative disorders known as transmissible spongiform encephalopathies or prion diseases. Human prion diseases occur in sporadic, genetic, and acquired forms [1]. The most frequently occurring is sporadic Creutzfeldt-Jakob disease (sCJD), accounting for approximately 85% of cases. Notably, in 1996, a novel form of CJD, termed variant Creutzfeldt-Jakob disease (vCJD), was discovered in the United Kingdom (UK), the cause of which has been linked to the consumption of beef products contaminated by the Bovine Spongiform Encephalopathy (BSE) agent [2].

vCJD cases are typically marked by an earlier age of disease onset and an extended disease duration compared to sCJD [3]. The clinical features of vCJD are reasonably stereotyped, with an initial phase typically dominated by psychiatric symptoms such as depression, delusions, and anxiety, followed by rapid development of neurological features including dementia, chorea, ataxia, and persistent painful sensory symptoms. [3, 4]. The diagnosis of vCJD has crucial implications for clinical management and public health due to the unique transmissibility of the disease. Definitive diagnosis relies on neuropathological confirmation through cerebral biopsy or necropsy; however, standard clinical diagnostic criteria have been established and validated, allowing for reasonably accurate diagnosis of most cases during life [4].

**Fig. 1:**
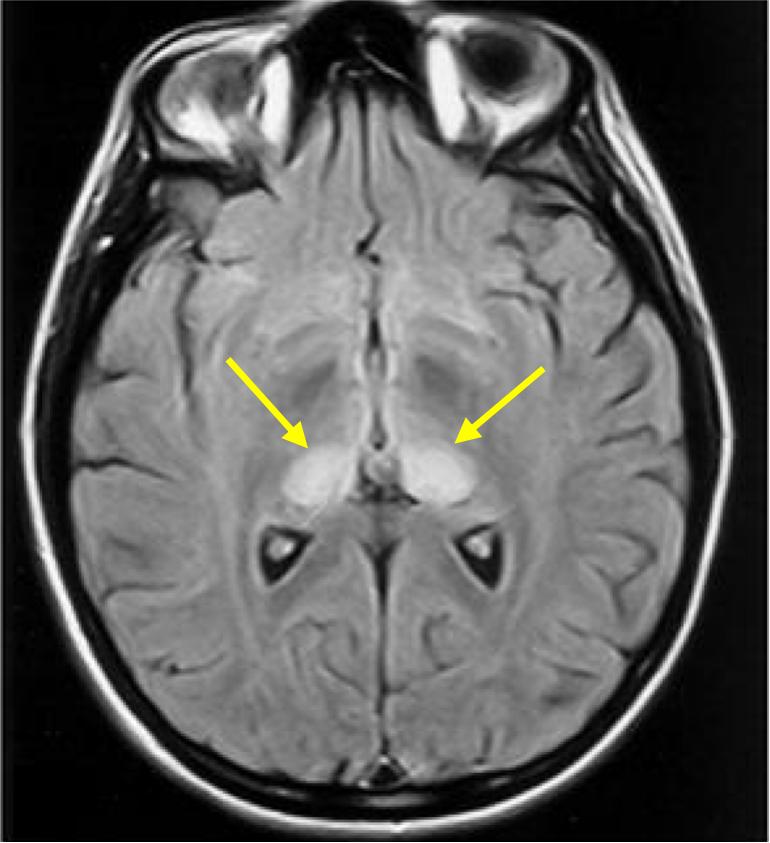
MRI brain in variant CJD - axial FLAIR section at the level of the basal ganglia demonstrating bilateral hyperintensity in the pulvinar nuclei of the posterior thalamus (arrows)

Magnetic resonance imaging (MRI) of the brain serves as a vital non-invasive investigation for diagnosing vCJD. Characteristic findings include symmetrical hyperintensity in the posterior thalamus (relative to the grey matter of the caudate head) known as the ’pulvinar sign’ [3–5] [Fig. 1]. This sign is rare in other forms of the disease [6–8] and has a reported sensitivity of 78-90% and a specificity of 100% for vCJD in the appropriate clinical context [4, 9, 10]. In addition to pulvinar signal changes, abnormalities have also been observed in the mediodorsal thalamic nucleus, caudate nucleus, putamen, and periaqueductal grey matter, while cortical changes are rare, in contrast to that observed in sCJD [11–13].

**Fig. 2:**
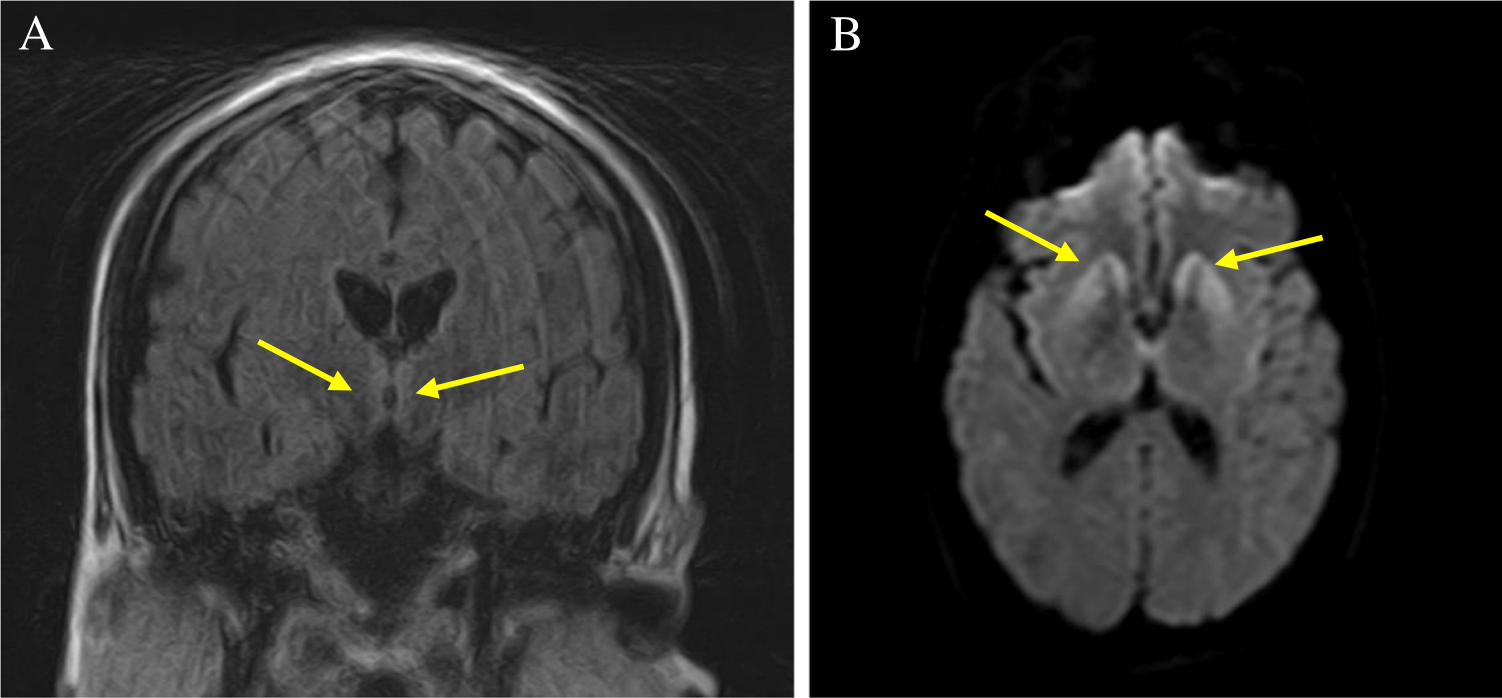
MRI brain of heterozygous (MV) variant CJD case. (**A**) Coronal FLAIR showing medial thalami hyperintensity only (arrows) and (**B**) Axial diffusion-weighted image demonstrating hyperintensity in the basal ganglia (arrows), medial thalami and bilateral insular cortices

To date, a total of 178 cases of vCJD have been identified in the UK, with an additional 55 cases from 11 other countries [14]. Genetic analysis conducted on 161 UK cases revealed that 160 were methionine homozygous (MM) at codon 129 in the prion protein gene (*PRNP*). This commonality may partly account for the shared MRI appearances seen among these cases, especially when contrasted with other prion diseases. The one remaining case with genetic analysis data was confirmed neuropathologically to be heterozygous (MV) at codon 129 [15]. The individual’s MRI depicted hyperintensities in the basal ganglia, mediodorsal thalami, and insular cortices on DWI, but not in the pulvinar nuclei, thus aligning more closely with sCJD radiological manifestations. The FLAIR images in this case were compromised by movement artifact, limiting their diagnostic efficacy. Nevertheless, they failed to demonstrate clear pulvinar signal change [Fig. 2].

The pivotal research assessing MRI appearances in vCJD established FLAIR imaging as the superior sequence in identifying the pulvinar sign, compared to T2-weighted or Proton Density (PD) weighted imaging [10]. Despite this, the study had a notable scarcity of cases featuring DWI (2 out of 110 MR examinations), which was likely due to DWI being a relatively novel sequence during the peak incidence of vCJD, leading to inconsistent availability across UK hospitals. Consequently, while DWI has risen to prominence as the most sensitive sequence for detecting disease-associated hyperintensities in sCJD [16–21], the literature remains limited regarding its appearances in vCJD, with limited discussion [22, 23] and no concentrated investigation into whether it could offer superior detection of pulvinar hyperintensity. Given the potential occurrence of additional heterozygous vCJD cases in the future, this study's primary aim is to examine the imaging characteristics and overall diagnostic sensitivity of DWI in the radiological diagnosis of vCJD.

## Methods

The National CJD Research and Surveillance Unit (NCJDRSU) has systematic information on all cases of CJD in the UK diagnosed since May 1990. This includes clinical data as well as copies of MRI brain scans in the majority of cases where this was performed. Using this database, MRI scans from patients with both probable and definite variant CJD with Diffusion Weighted Imaging (DWI) were identified for analysis. Serial scans, if available, were also analysed. Where scans were incomplete or had not been retained, copies were requested from the original referring hospital. MRI scanner parameters, including magnet strength (1.5/3Tesla etc) were not available from the data collected.

The scans were reviewed by a Neuroradiologist, experienced in the assessment of MRI in CJD, for the presence or absence of features associated with these diseases. The Neuroradiologist was aware of the suspicion of CJD but was blinded to the specific diagnosis (i.e. sporadic, variant etc). Scans were reviewed within a larger mixed cohort of CJD cases including neuropathologically confirmed sCJD and non-CJD controls.

As most probable cases of vCJD were classified as such on the presence of a supportive MRI (positive pulvinar sign), it was decided to analyse these cases separately to those confirmed neuropathologically so as not to confound results.

### MRI sequences

Only MRI scans containing DWI were included for the study. Of these, the majority contained T1/T2-weighted sequences and fluid-attenuated inversion-recovery (FLAIR) sequences. 

### Image quality

Image quality was subjectively scored based on the presence and degree of movement artifact and the ability to clearly delineate the boundaries between grey-matter and white-matter structures. DWI is less affected by movement artifact compared to other sequences, owing to a shorter acquisition time. Therefore, image quality was assessed across all images and not just DWI alone. Scan quality was graded on a scale of 1 to 6, with 5 and 6 considered to be of non-diagnostic quality (1=excellent, 2=good, 3=average, 4=sufficient, 5=insufficient, 6=poor).

### MRI analysis

MRI scans were visually assessed for the presence or absence of hyperintensity observed within the cerebral cortex, caudate heads, putamen, and posterior and dorsomedial thalamic nuclei on both FLAIR and DWI sequences. The cortical areas assessed included frontal, cingulate, temporal, parietal, and occipital regions. The sequences were assessed independently, and each scored as either positive or negative for the presence of the pulvinar sign, defined as bilateral hyperintensity within the pulvinar nuclei of the thalamus relative to that of the caudate head. The MRI was also classified according to the likely CJD subtype (variant/sporadic/undetermined) based on the radiological findings. The results of this classification are presented separately.

### Statistical analysis

Descriptive statistics were used to summarise the MRI findings, including frequencies and percent- ages for categorical variables. McNemar’s test was employed to assess and compare the sensitivities of FLAIR and DWI in the detection of the pulvinar sign.

## Results

### Variant CJD cases demographics and clinical data

A total of 178 cases of definite or probable variant CJD were identified in the UK, including 123 definite and 55 probable cases. MRI brain scans were performed in 176 cases, of which 27 had DWI. Two cases were excluded due to poor DWI quality. The remaining 25 cases included 15 neuropathologically confirmed cases and 10 probable cases, with the latter group predefined based on an appropriate clinical history and supportive MRI scan or tonsil biopsy or both (Table 1).

**Table 1:** Clinical details of probable vCJD cases including supportive diagnostic investigation

Case no	Age	Disease duration (months)	Positive Pulvinar sign	Positive tonsil biopsy
1	25	13	Yes	N/A
2	33	15	Yes	N/A
3	34	11	Yes	N/A
4	29	12	Yes	N/A
5	23	13	**No**	**Yes**
6	20	6	Yes	N/A
7	47	45	**No**	**Yes**
8	39	16	Yes	Yes
9	22	22	Yes	N/A
10	46	12	Yes	N/A

The date in which the MRI scan was performed ranged between November 1999 and August 2015. Most scans were Digital Imaging and Communications in Medicine (DICOM) data, with eight scans on plain film. A total of 34 scans were available from the 25 cases; seven with serial images (six had two images and one had four). The median disease duration was 13 months (range 6 - 45) with median age of disease onset 33 (range 15 - 74).

Most scans (29 out of 34) were of good or average diagnostic quality, with minimal motion artefact. Four scans were graded as sufficient, and one case was graded as insufficient due to a combination of movement artefact and lack of FLAIR sequences.

### Sporadic CJD and non-CJD cases demographics and clinical data

The vCJD MRI scans were reviewed within a larger mixed cohort of CJD scans consisting of neuropathologically confirmed sCJD (definite sCJD) and controls where a diagnosis of CJD was suspected in life but with an alternative diagnosis confirmed by autopsy (definite non-CJD cases). Only cases where both FLAIR and DWI sequences were available (sCJD n=188 and non-CJD cases n=19) were included for analysis in this study. All were DICOM data of good or average quality performed between January 2010 and January 2016.

**Fig. 3:**
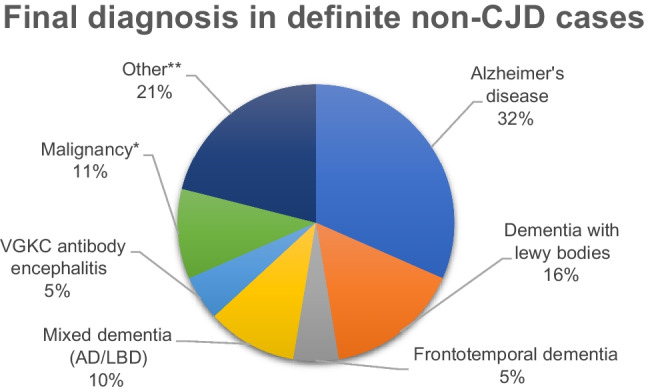
Diagnosis of non-CJD cases confirmed on autopsy * Malignancy = one case with Diffuse B cell Lymphoma and one with Chronic Myeloid leukaemia ** Other = four cases with no evidence of CJD at autopsy, one with non-necrotising granulomatous disease and one with multi-focal necrotising granulomatous disease. (VGKC - Voltage gated potassium channel, AD - Alzheimer’s disease, LBD - Lewy body dementia)

The median disease duration in sCJD was 4.53 months (0.87 - 62.03) compared to 8 months (2.13 - 59.87) in controls with median age of disease onset 68 (25-85) and 73.5 (58-89) years respectively. Codon 129 data was available in 185 sCJD cases (MM=114, MV=32, VV=39) and in 7 controls (MM=4, MV=0, VV=2). Figure 3 shows the final diagnosis in non-CJD cases.

Figure 3 illustrates the final diagnosis in the non-CJD patients.

### MRI analysis in probable vCJD cases

#### MRI findings in probable cases

**Fig. 4:**
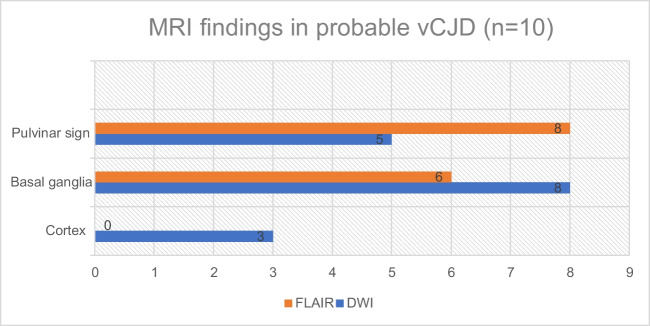
MRI findings in probable cases of vCJD

In the 10 probable cases of vCJD, eight had positive pulvinar signs on FLAIR images, while two were negative (Fig. 4). The latter two cases had positive tonsil biopsies. In comparison to FLAIR, only five cases had pulvinar signs identified on DWI, all of which were positive on FLAIR. No cases showed positive DWI when the corresponding FLAIR was negative. Six cases demonstrated basal ganglia hyperintensity on FLAIR compared to eight on DWI. Cortical high signal was identified in three cases, only on DWI.

#### MRI diagnosis in probable cases

**Fig. 5:**
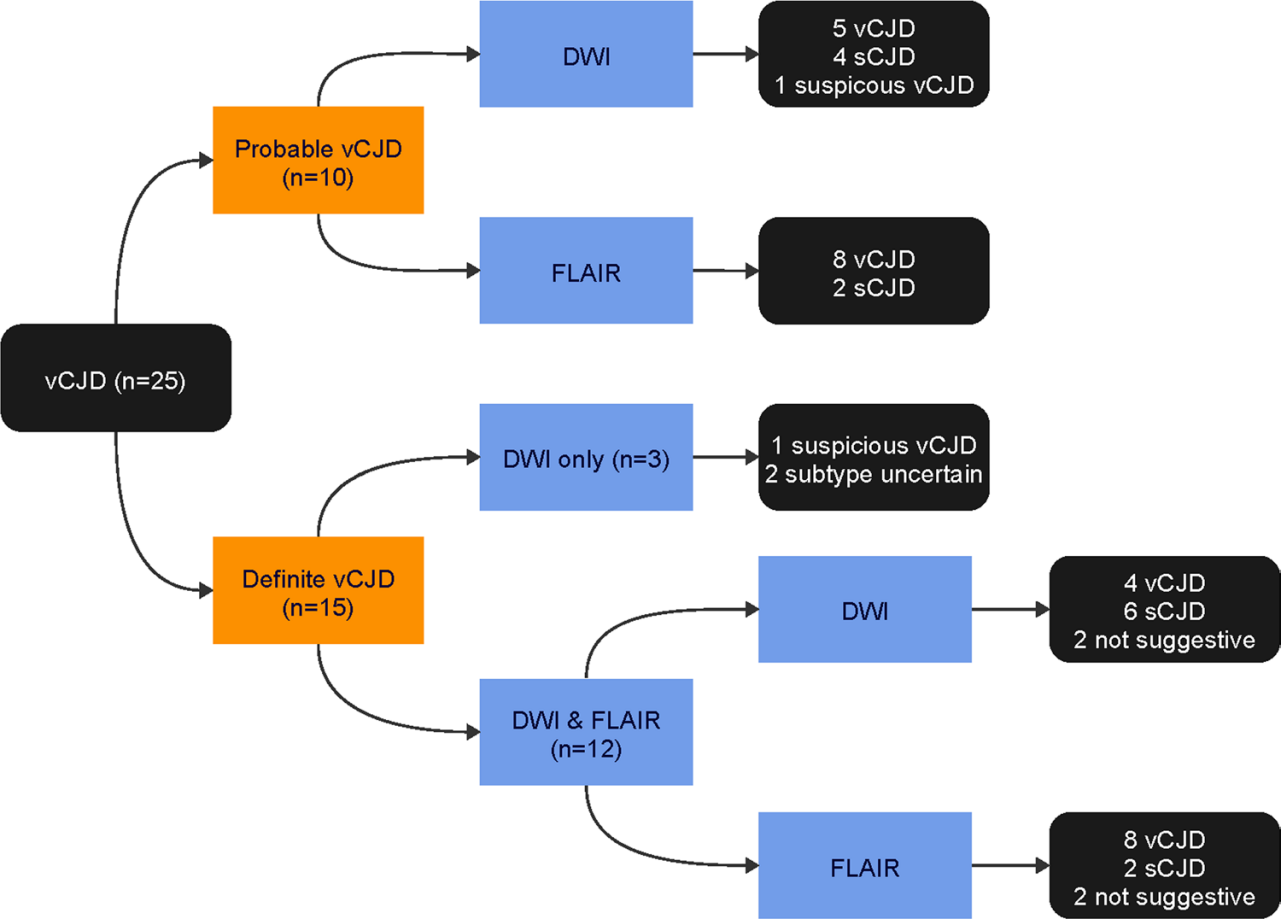
MRI diagnosis in probable and definite cases of vCJD

Based on FLAIR images, eight scans were classified radiologically as variant CJD due to the presence of the pulvinar sign, and two as sporadic CJD due to basal ganglia hyperintensity and absent pulvinar signs (Fig. 5). On DWI, five scans were classified as variant CJD due to positive pulvinar signs (all of which were positive on FLAIR), four as sporadic CJD with absent pulvinar signs, and one as suspicious of variant CJD with absent pulvinar but isolated mediodorsal thalamic hyperintensity.

### MRI Analysis in Definite vCJD Cases

#### MRI findings in definite cases

**Fig. 6:**
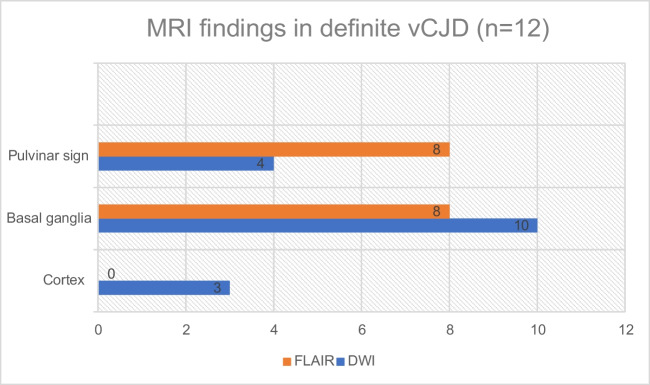
MRI findings in definite cases of vCJD (*with both FLAIR and DWI)

**Fig. 7:**
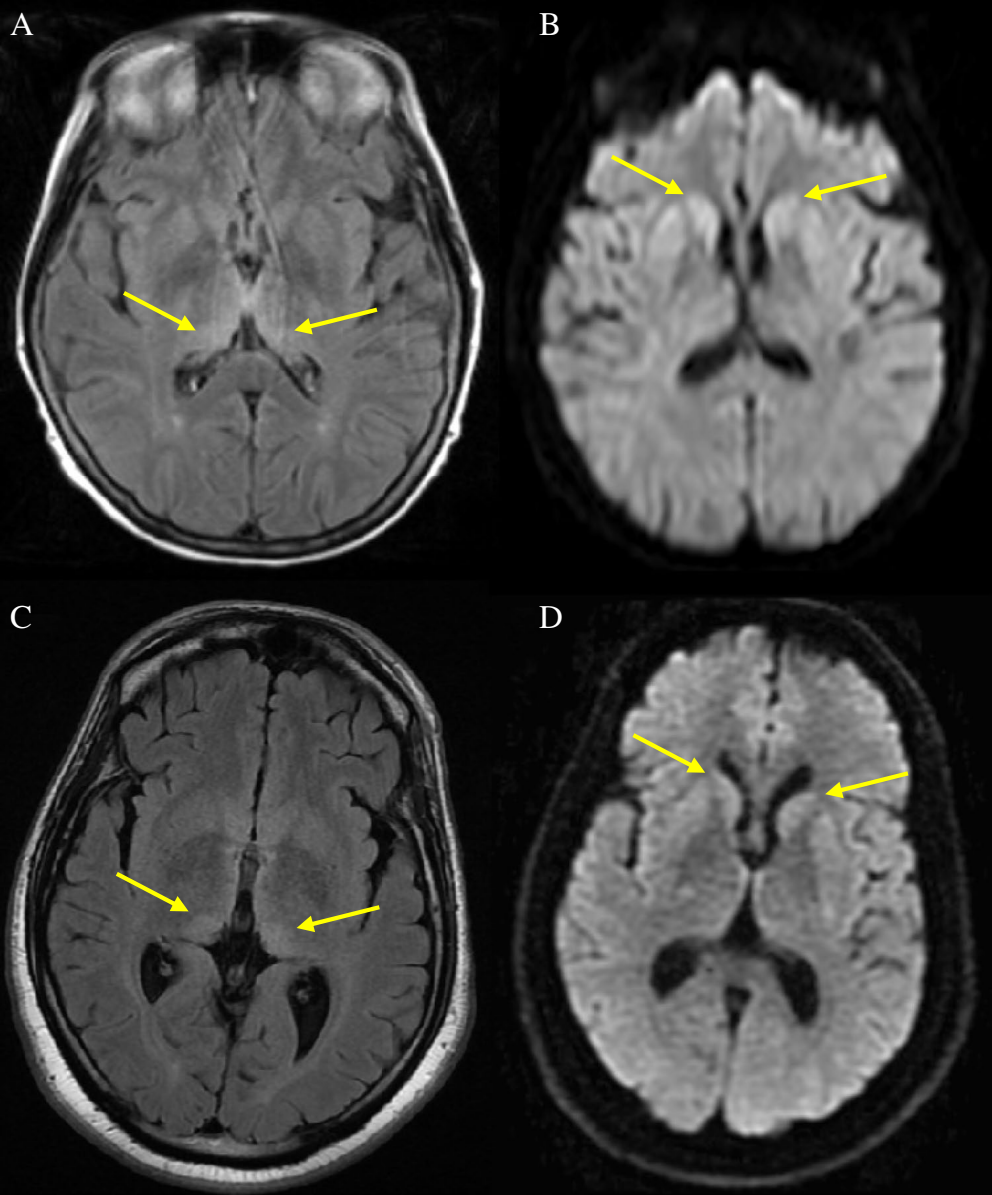
MRI findings in two neuropathologically confirmed cases of vCJD(**A+B**) and (**C+D**) Axial FLAIR images in both cases (**A** and **C**) demonstrating positive pulvinar signs, and associated dorsomedial thalamic high signal, with the corresponding DWI (**B** and **D** respectively) showing more conspicuous high signal in the basal ganglia in the absence of clear pulvinar signal abnormality

Of the 15 neuropathologically confirmed cases of vCJD, 12 had both FLAIR and DWI sequences (Fig. 6), and three had DWI alone. Among the 12 cases with both sequences, eight had positive pulvinar signs on FLAIR and four had negative signs. In corresponding DWI sequences, four had positive pulvinar signs and eight had negative signs (Fig. 7). No instances showed positive DWI when the FLAIR was negative.

Basal ganglia high signal was present in eight cases on FLAIR compared to 10 on DWI. Cortical signal hyperintensity was not evident on any FLAIR sequences but was present in three cases on DWI. Among the three cases with only DWI available, all had basal ganglia and thalamic high signal, one had associated cortical signal hyperintensity, and all were negative for the pulvinar sign.

#### MRI diagnosis in definite cases

**Fig. 8:**
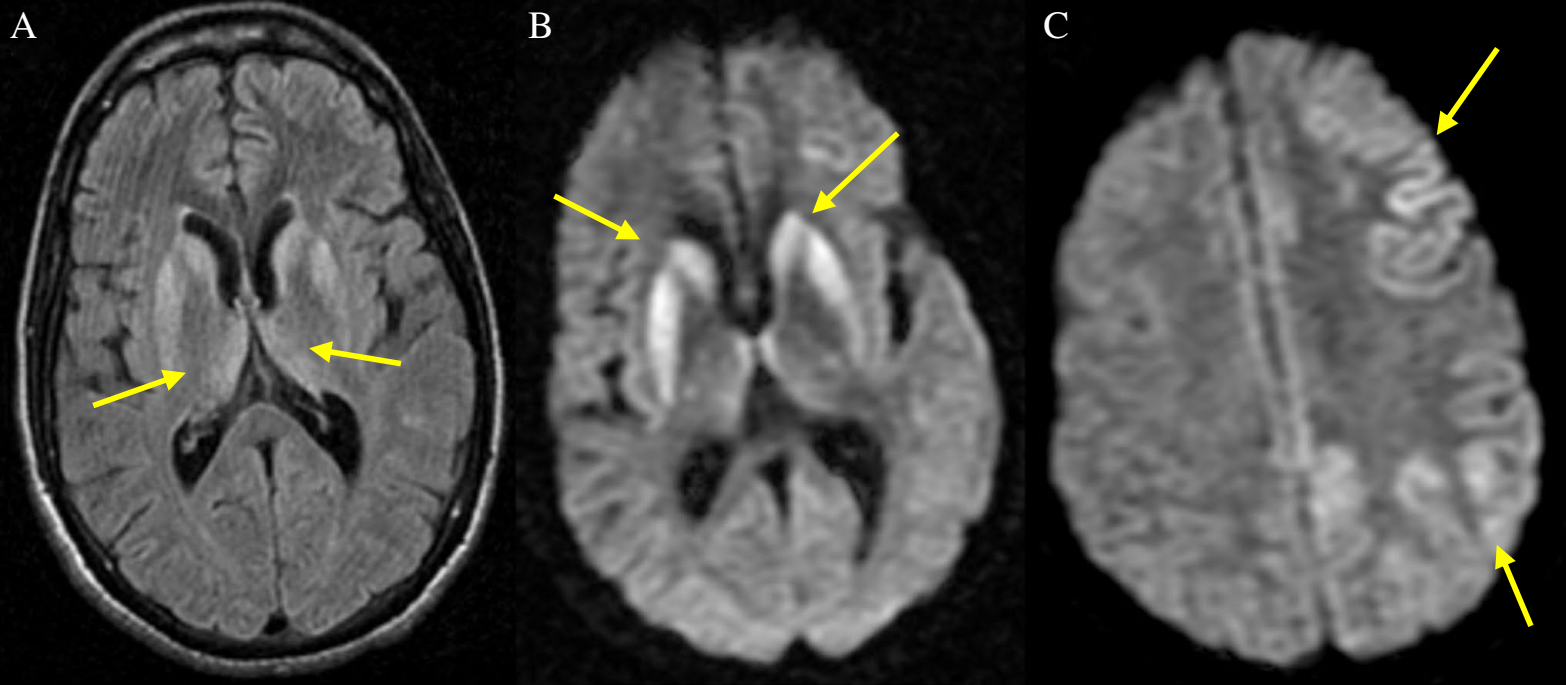
MRI in a confirmed case of vCJD with appearances more characteristic of sporadic CJD (**A**) Axial FLAIR showing mediodorsal and pulvinar high signal (arrows) of equal intensity to basal ganglia. (**B**) DWI showing more conspicuous high signal in basal ganglia (arrows) with less marked dorsomedial thalamic abnormalities and (**C**) associated left parietal and frontal cortical hyperintensity (arrows)

In the 12 cases with both FLAIR and DWI sequences, eight were classified radiologically as vCJD on FLAIR, two as sCJD, and two as non-suggestive (Fig. 5). In the corresponding DWI, four cases had appearances of vCJD, six sCJD (Fig. 8), and two were non-suggestive. Of the three cases with DWI only, two were classified as CJD of uncertain subtype and one as suggestive of vCJD but not diagnostic.

### Cortical hyperintensity in vCJD

Among the 10 probable cases, four were observed to have cortical hyperintensities (three when excluding the cingulate gyrus) with a median two cortical regions involved, ranging between 1 -4. In decreasing frequency, the regions involved were; parietal (29%, n=2), cingulate (29%, n=2), frontal (14%, n=1), insular (14%, n=1), and occipital (14%, n=1).

Of the 15 definite cases, six had cortical hyperintensities (three when excluding the cingulate gyrus), with a median of two cortical regions involved, again ranging between 1 - 4. In decreasing frequency, the regions involved were: cingulate (56%, n=5), insular (22%, n=2), frontal (11%, n=1), and parietal (11%, n=1).

### Sporadic CJD and non-CJD cases

#### MRI findings in sporadic CJD

Basal ganglia high signal was found in 138 sCJD cases (73.4%) on DWI compared to 71 (37.8%) on FLAIR. Cortical high signal was observed in 84% of cases on DWI compared to 42.1% on FLAIR. 152 of these cases had associated basal ganglia involvement and in 51 cases the cortical high signal was identified in isolation.

**Fig. 9:**
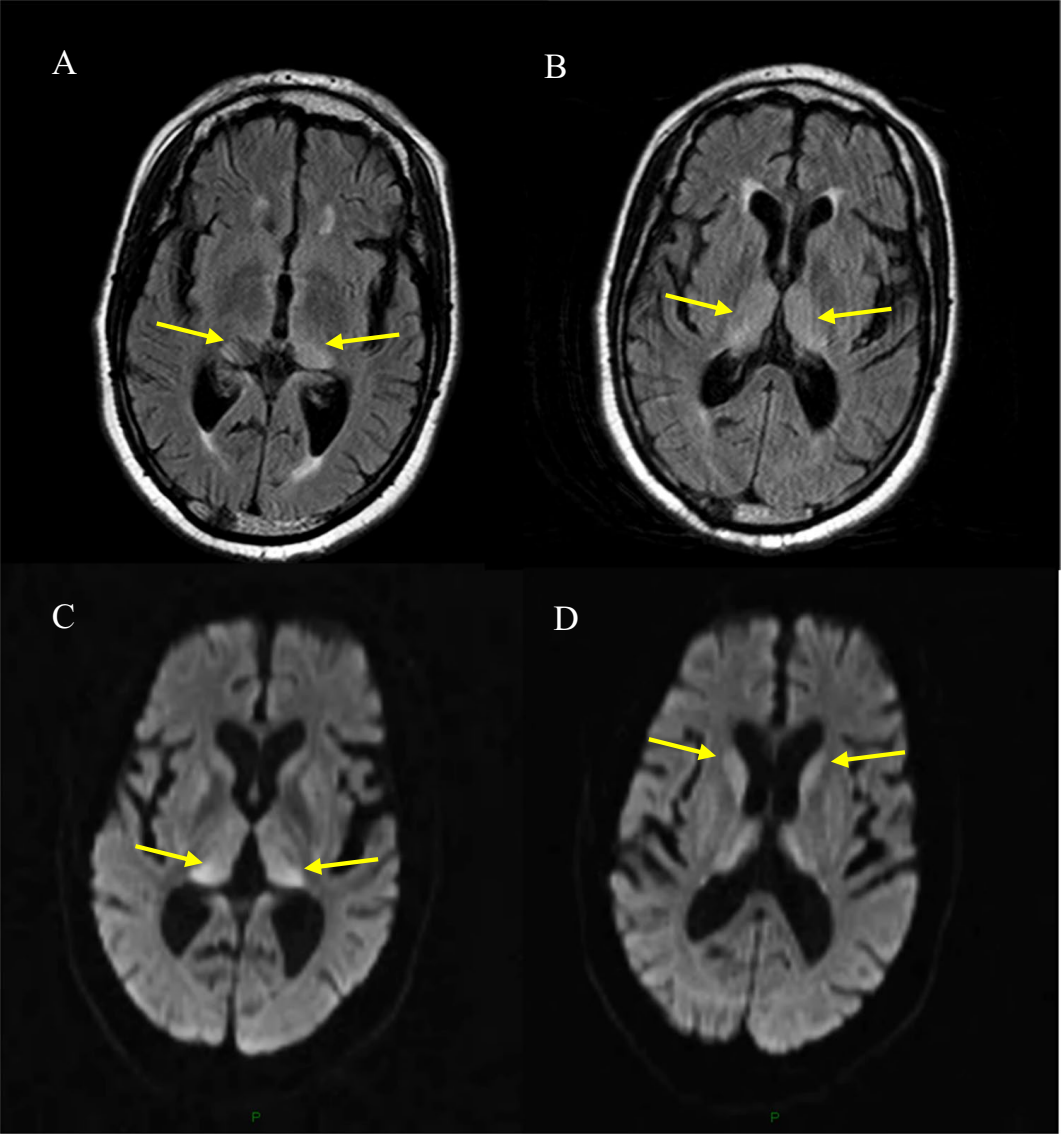
Positive pulvinar sign in definite MV2 sCJD patient. Axial FLAIR sequences (**A+B**) showing positive pulvinar sign (**A**) (arrows) with (**B**) associated dorsomedial thalamic high signal (arrows). DWI sequences (**C+D**) demonstrating more conspicuous pulvinar high signal (arrows) with (**D**) associated dorsomedial thalamic and bilateral caudate head high signal (arrows)

There were two sCJD cases with pulvinar signs (as defined above); one with a pulvinar sign on FLAIR and DWI and the other only on FLAIR imaging only (Fig. 9). Both were MV2 cases and both had associated basal ganglia high signal in the absence of cortical involvement.

### MRI findings in non-CJD cases

**Fig. 10:**
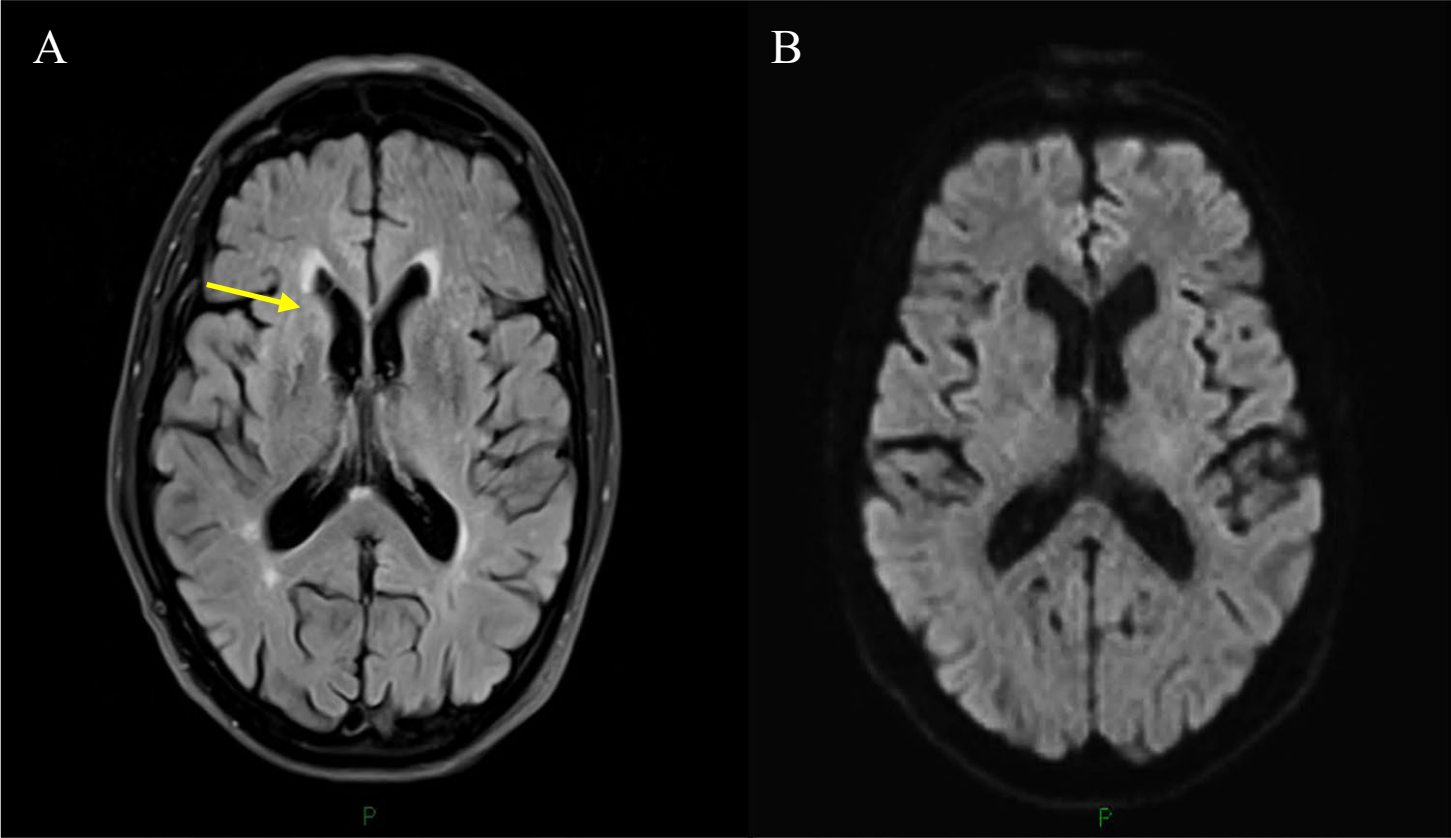
Axial MRI brain of a 74-year-old with autopsy confirmed Lewy body dementia. The FLAIR (**A**) shows subtle right caudate head signal abnormality with no associated high signal on DWI (**B**)

Basal ganglia high signal was found in 2 non-CJD cases (11.1%), one with Lewy body dementia and one with mixed Alzheimer’s and Lewy body pathology. In both cases, the caudate heads were involved (one with symmetrical involvement) with no corresponding high signal on DWI (see Fig. 10).

Cortical high signal was found in six (22%) non-CJD cases (two on FLAIR only and four DWI only). None of these cases had associated basal ganglia or thalamic high signal. The cortical regions involved were limited to one region in 3 cases (multifocal necrotising granulomatous disease, dementia with Lewy body and one case with no evidence of CJD at autopsy) and to two regions in 3 cases (mixed Alzheimer’s and Lewy body pathology, autoimmune encephalopathy and Alzheimer’s disease). Only two controls demonstrated cortical signal abnormalities when excluding regions prone to signal artefact (frontal, cingulate and insular cortices).

**Fig. 11:**
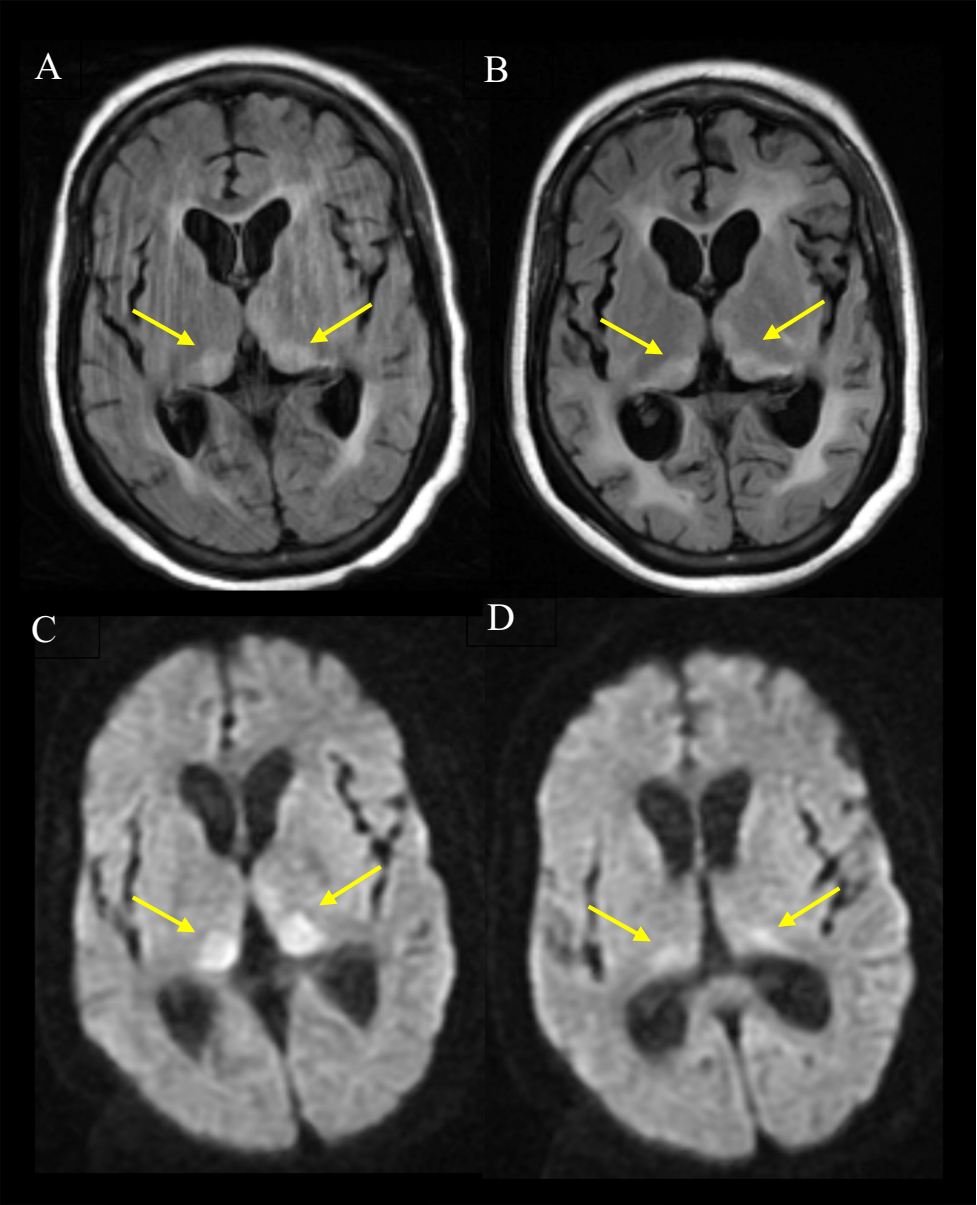
Serial MRI scans (**A+C**) and follow up scan at 6 months (**B+D**) of neuropathologically confirmed case of Alzheimer’s disease with positive pulvinar sign. Axial FLAIR sequences (**A+B**) showing positive pulvinar sign (**A**) (arrows) with (**B**) associated dorsomedial thalamic high signal on repeat scan (arrows). DWI sequences (**C+D**) demonstrating more conspicuous pulvinar high signal (arrows) with (**D**) less marked thalamic hyperintensity on follow up scan (arrows)

Thalamic signal abnormality was present in two non-CJD cases. One was proven to have chronic myeloid leukaemia on autopsy, involving the dorsomedial and pulvinar regions on FLAIR imaging only, with no associated signal abnormalities on DWI. The imaging was atypical for vCJD in that there was associated periventricular and pontine high signal. The other case was a patient with Alzheimer’s disease that had pulvinar high signal on FLAIR and DWI. On repeat imaging, performed 6 months later, the thalamic high signal extended into the mediodorsal thalamus with less marked signal change on DWI and progressive atrophy and white matter changes (see Fig. 11).

### Sensitivity and specificity of the pulvinar sign in vCJD

In the probable and definite vCJD cases, where both FLAIR and DWI sequences were available, FLAIR sequences identified the pulvinar sign in 16 of the 22 cases whilst DWI identified it nine cases (sensitivity of 73% vs 41% respectively, p-value<0.001).

In this studies analysis of cases, the pulvinar sign demonstrated an overall specificity of 98.9 % in the radiological diagnosis of vCJD.

### Cases with serial imaging

#### Probable cases with serial scans

Four probable cases had serial imaging; three with two scans and one with four. Two of the four probable cases were positive for the pulvinar sign on initial scans and subsequent scans (based on FLAIR, with one having corresponding pulvinar sign on DWI in both). The other two cases with serial scans were both negative for the pulvinar sign on initial imaging. One of these cases had a positive pulvinar sign detected on subsequent imaging (FLAIR only). The other case had four serial scans that were all negative for the pulvinar sign.

#### Definite cases with serial scans

In definite cases, three neuropathologically confirmed cases had two serial scans each. Two of these cases were positive for the pulvinar sign on FLAIR images (one having a corresponding pulvinar sign on DWI) on both the initial and subsequent scans. Of these two cases, one was classified as vCJD based on image appearance alone, and the other as sporadic, due to the presence of cortical hyperintensities on DWI (bilateral cingulate and insular). The latter case changed radiological classification on the subsequent scan to vCJD, due to a positive pulvinar sign in the absence of previously observed cortical hyperintensities. The remaining case, which was negative for the pulvinar sign, was classified as non-suggestive of CJD on initial and subsequent examination. This was a blood transfusion-related case, and the initial scan showed bilateral cingulate hyperintensities, with the subsequent scan showing bilateral cingulate and unilateral insular hyperintensities.

### Timing of MRI in relation to disease duration

**Fig. 12:**
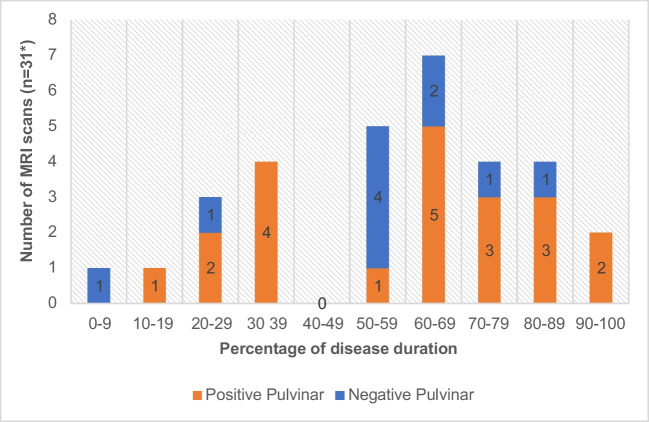
Graph showing timing of FLAIR imaging in relation to disease duration in both probable and definite cases(*including serial scans)

**Fig. 13:**
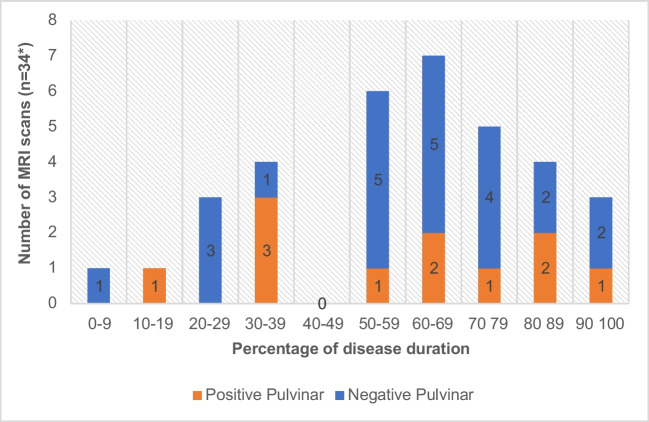
Graph showing timing of DWI in relation to disease duration in both probable and definite cases(*including serial scans)

Negative MR imaging occurred at most stages in the disease process on both FLAIR and DWI (Figs. 12 and 13).

## Discussion

Our study offers a unique perspective on the application and effectiveness of diffusion-weighted imaging in diagnosing variant Creutzfeldt-Jakob Disease (vCJD). Contrary to the general expectation, DWI imaging demonstrated an inferior sensitivity in detecting the pulvinar sign compared to FLAIR (sensitivity of 41% vs 73% respectively, p-value <0.001). Interestingly, on DWI, the pulvinar hyperintensity was typically less pronounced, whereas hyperintensities in the anterior basal ganglia and cortical areas were more prominent, resulting in image appearances more commonly associated with sporadic CJD. Consistent with previous research, our findings reaffirm the high specificity of the pulvinar sign, in the correct clinical context, in the diagnosis of vCJD and its rarity in sCJD. The study also noted that cortical hyperintensity was a less common feature in vCJD unlike the extensive cortical involvement typically observed in sCJD. Both FLAIR and DWI presented negative imaging, that is absence of the pulvinar sign, across varying stages of the disease process.

The underlying cause for DWI hyperintensity in vCJD is not fully elucidated, but likely is the result of a combination of microvaculolation resulting in true diffusion restriction, and gliosis due to microglial and astrocyte activation causing prolonged T2 values [23]. In sCJD it has previously been shown that the DWI restriction in sporadic CJD is largely due to microvacuolation, and that increased vacuolation occurs with increasing disease course [24]. This has not been examined in vCJD. Considerable increases in gliosis have been noted pathologically particularly in the pulvinar nuclei in vCJD compared with sCJD [25]. T2 hyperintensity arising from this would be expected to produce hyperintensity on both FLAIR and DWI b1000 mean diffusivity images and may explain the increased FLAIR conspicuity relative to DWI imaging. In vCJD. The ADC data from these vCJD cases was often unavailable or too heterogeneous to undertake accurate ADC measurement. It's important to recognise that pathology showcases the disease's state at its endpoint, at death. In contrast, MRI changes are observed earlier in the disease's progression. Thus, directly comparing pathology to MRI findings can be challenging due to the temporal disparity in their respective stages of observation.

An intriguing observation was that the neuropathologically confirmed heterozygous vCJD case exhibited DWI appearances similar to those observed in homozygous cases. The defining features were more substantial hyperintensities in the basal ganglia and minimal cortical involvement, with less conspicuous pulvinar signal change. Further corroborating our study, a recent homozygous vCJD case reported in France, attributed to unintended occupational exposure to prion-contaminated BSE material, showcased MRI features that mirror our findings [26]. In this case, the pulvinar sign was identifiable on FLAIR imaging. However, the DWI displayed more obvious signal hyperintensity in the anterior basal ganglia. This pronounced anterior basal ganglia signal was of at least equal intensity to the pulvinar hyperintensity, rendering the identification of the pulvinar sign challenging on DWI. There was no reported cortical hyperintensity.

**Fig. 14:**
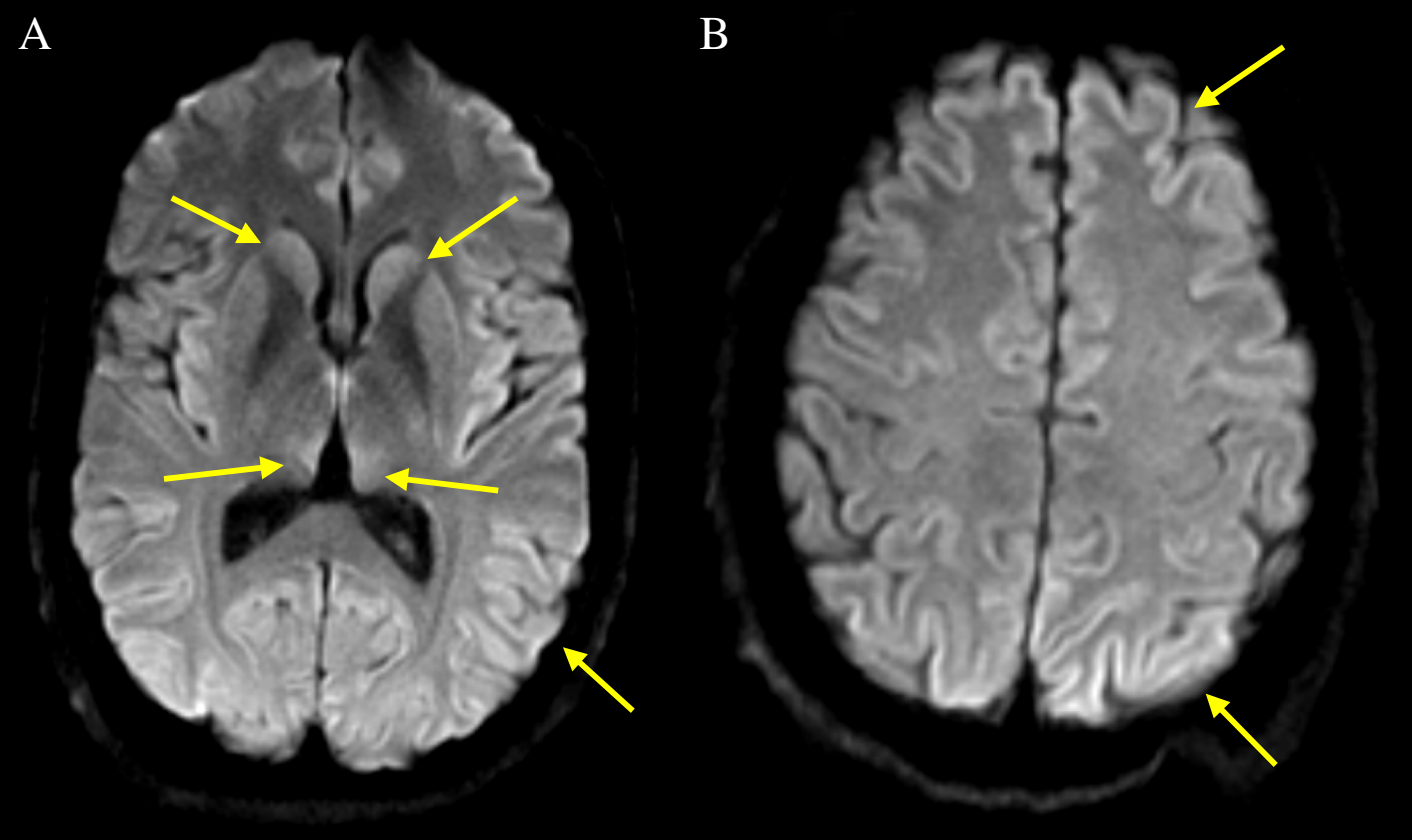
Axial DWI sequences in 'possible’ MV vCJD casedemonstrating (**A**) basal ganglia and pulvinar hyperintensities (arrows) with associated widespread cortical hyperintensities (**A** and **B**)

The above findings gain additional significance when compared to a 2009 report by Kaski et al of a suspected diagnosis of vCJD in an individual with MV genotype, who died without post- mortem [27]. The MRI of this case revealed the pulvinar sign on FLAIR imaging, yet it diverged notably from the neuropathologically confirmed MV vCJD and other MM cases referenced above, with the DWI displaying widespread cortical hyperintensity encompassing bilateral frontal, cingulate, parietal, and occipital regions, alongside basal ganglia and thalamic involvement (**Fig.** 14). The divergence of DWI appearances in this case potentially amplifies the complexity of our understanding of vCJD radiological appearances. However, due to the lack of post-mortem examination, we can only speculate about the nature of this case. This draws attention to the need for comprehensive investigations in all suspected vCJD cases, and on-going disease surveillance, to understand fully the spectrum of neuroimaging features associated with this condition.

In a broader perspective, there is substantial overlap in the MRI appearances of grey matter structures in different forms of CJD, particularly so in sCJD [18, 20, 21, 28]. Meissner et al assessed MRI signal profiles in sCJD based on protein subtype [28]. In their study, MV1 cases often showed cerebral cortex and basal ganglia involvement. In the MV2 cases, the basal ganglia and thalamus were characteristically affected, with the thalamic high signal observed frequently within the pulvinar area and the pulvinar sign being present in three patients.

Of course, in clinical practice a diagnosis of CJD is not based on the MRI findings in isolation. Rather, the MRI requires to be interpreted alongside the appropriate clinical context such as age of onset, clinical features and disease duration. Iatrogenic CJD can often be distinguished based on previous exposure (e.g. human pituitary-derived growth hormone/human dura mater grafts) and familial CJD on the basis of genetic analysis. The difficultly arises when clinical features overlap, particularly so in sporadic and variant CJD. Initially, it was anticipated that MRI would offer a reliable distinction between these forms, but it appears not to be the final arbitrator in all cases. Although CSF RT-QuIC has emerged as a highly sensitive and specific test for sCJD, the assay in its current form does not amplify the *PrPSc* in vCJD [29, 30]. CSF protein misfolding cyclic amplification (PMCA) on the other hand, appears to be a promising pre-mortem test for vCJD and may differentiate patients with heterozygous vCJD from those with sCJD [31].

## Conclusion

In essence, our study reveals that FLAIR imaging is superior to DWI in terms of identifying the pulvinar sign, while DWI tends to highlight hyperintensities in the basal ganglia and cortical areas more distinctly, creating image appearances more akin to sporadic CJD. Hence, it is crucial to approach DWI interpretation with caution when considering a diagnosis of vCJD.

In light of our research, we advocate an adjustment to the current diagnostic criteria for vCJD, emphasising the use of axial FLAIR imaging to accurately determine the presence or absence of the pulvinar sign. This shift could significantly enhance the diagnostic accuracy for vCJD, thereby deepening our understanding of its distinctive neuroimaging manifestations.

## Limitations

Our study is open to several criticisms. A methodological limitation was that MRI scans were performed under different conditions on many different MR imaging platforms across the country, which resulted in greater technical heterogeneity of the imaging. It is also limited by the small number of cases with DWI as well as being generally limited by the quality of DWI sequences during the study time period. Of the cases that had DWI, a proportion were hard copy images which introduces several uncontrolled limitations in image quality compared to digital data, such as the inability to change window settings etc. In addition to these factors, the MRIs were visually assessed when examining for areas of high signal, rather than the quantitative measures used in other imaging studies. The identification of MRI abnormalities in CJD however, are based on visual assessment as part of the diagnostic process and so this study’s analysis is applicable to that encountered in clinical practice.
